# A graphene/Si Schottky diode for the highly sensitive detection of protein

**DOI:** 10.1039/c9ra03765a

**Published:** 2019-06-24

**Authors:** Ali Akbar Noroozi, Yaser Abdi

**Affiliations:** Nanophysics Research Laboratory, Department of Physics, University of Tehran Tehran Iran y.abdi@ut.ac.ir

## Abstract

Herein, a graphene/Si-based device was introduced for bovine serum albumin (BSA) sensing. In this study, it is shown that the Schottky junction at the interface of graphene/Si is highly sensitive to BSA under UV light exposure. The reverse bias current of the junction, which is sensitive to UV light, changes under exposure to BSA at different concentrations. By UV light absorption, we showed that the addition of the BSA solution to the junction affected the output characteristic of the fabricated device. Moreover, the output characteristic of the device shows that the device can be considered as a self-powered detector that would reduce the need for batteries. The results obtained in this study would open up a way towards the fabrication of an on-chip biosensor for the sensing of biological agents such as BSA.

## Abstract 2

Herein, a graphene/Si-based device was introduced for bovine serum albumin (BSA) sensing.

## Graphical abstract

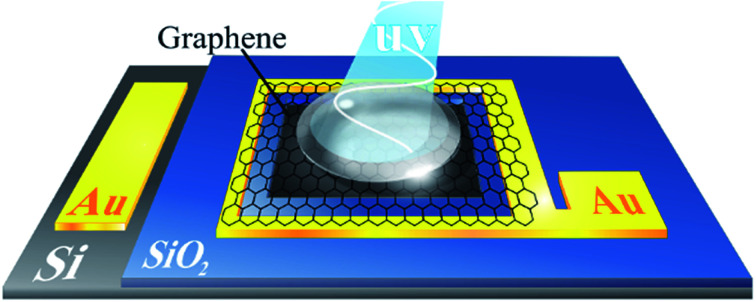

## 1. Introduction

Graphene, a two-dimensional material, has been widely used for the fabrication of chemical sensors, photodetectors and biosensors.^1–3^ The extremely high surface-to-volume ratio makes graphene-based sensors highly sensitive to the changes in the chemical composition of an environment or biomass.^4–7^ Graphene has been used as a platform for the detection of different types of biomacromolecules such as DNA and proteins.^8–11^ Among biomacromolecules, serum albumin is the most abundant blood protein and essential for the growth of activated human lymphocytes.^12^ Consequently, the monitoring of serum albumin concentration would be useful. Because of its structural homology with human serum albumin, bovine serum albumin (BSA) is an appropriate protein to be used as a model protein for investigations and studies. The determination of serum albumin concentration is a critical part of protein studies. Therefore, some studies focusing on the quantification of the amount of protein present in a sample using efficient and low-cost methods have been reported.^13,14^ The common method for the determination of BSA concentration is based on the detection of UV absorbance of the samples. Most proteins exhibit a distinct ultraviolet light absorption maximum at 280 nm due to the presence of tyrosine and tryptophan. Since the tyrosine and tryptophan contents of various enzymes vary within narrow limits, the absorption peak at 280 nm has been used as a rapid and sensitive measure of protein concentration.^15^ In this method, the ratio of the absorbance at 280 nm for the target samples *versus* the absorbance of the sample at known concentration is measured. This method has moderate sensitivity with high material requirement. To reduce the material requirement and increase the sensitivity, it is necessary to focus on the on-chip measurements. In this study, we report the on-chip detection of BSA using a graphene/Si-based device.

In recent years, the graphene/Si junction has been utilized for the fabrication of highly sensitive photodetectors with sensitivities as high as 1–10 A W^−1^.^16–19^ Due to its wide range of absorption, graphene/Si is a promising candidate for photo-detection from the UV to the IR range.^20,21^ Recently, we have reported the fabrication of highly sensitive broadband photodetectors based on the vertical Schottky junction of graphene/Si.^22^ On the other hand, graphene/Si-based diodes have been reported to be utilized for chemical and glucose sensing. Sakr *et al.* have utilized graphene-Schottky junction for non-enzymatic glucose sensing. Their sensor shows a linear response to glucose concentration in the range from 0 to 15 mmol L^−1^ with the detection limit of 0.5 mmol L^−1^.^23^ It has been shown that chemical adsorption on graphene sheets changes the electronic properties of graphene. Consequently, it is possible to modify the electrical characteristics of the graphene/Si junction *via* chemical adsorption. Based on this idea, Kim *et al.* have reported a graphene/Si-based diode sensor in which the chemical agent has been directly applied onto the graphene sheet using a pipette.^24^

In this study, we report the fabrication of a graphene-based biosensor for the sensing of BSA to take advantage of both the optical and the electrical properties of the graphene/Si Schottky junction. This proposed sensor worked under exposure to UV light, and the junction photocurrent was plotted *versus* the serum albumin concentrations. Using this procedure, BSA sensing could be easily and quickly carried out on a chip without the need of bulky equipment.

## 2. Experimental

**Fig. 1:**
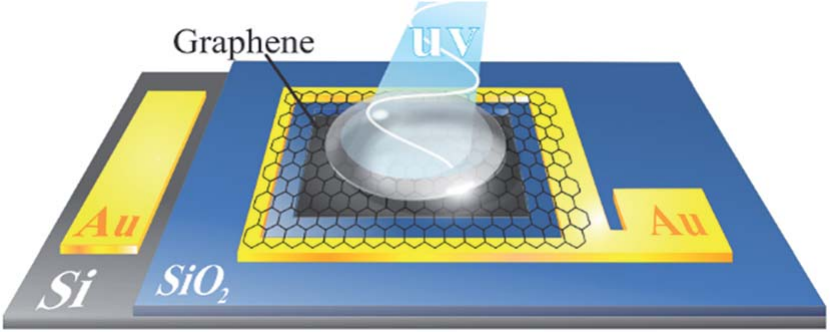
Schematic showing the structure of the fabricated graphene-based BSA sensor. The graphene sheet is connected to the surrounding Au electrode, and the middle part of the sheet is connected to the Si substrate.

The device fabrication process was started by cleaning a lightly doped (1–10 Ω cm) n-type silicon wafer using the RCA#1 solution. The cleaned wafer was then coated with a SiO_2_ layer using a thermal oxidation technique at 1200 °C. A 3 × 3 mm^2^ window in the SiO_2_ layer was then patterned using standard photolithography and then removed by the buffered hydrofluoric acid solution to obtain the desired pattern for the formation of the diode structure, as schematically shown in Fig. 1. The Ti/Au (20/80 nm) layer was coated on the Si/SiO_2_ structure and then patterned by photolithography to form contact electrodes (see Fig. 1). To transfer the graphene sheet on the Si substrate, monolayer graphene was coated on Cu foils (Graphenea Company, Spain) by poly methyl methacrylate (PMMA) using the spin coating method. The procedure was then followed by the dissolution of the Cu foil in ferric chloride and subsequent attachment of PMMA-coated graphene on the Si/SiO_2_ structure. Careful transfer of the graphene/PMMA sheet ensured that the sheet was connected to the surrounding Au electrode, as illustrated in Fig. 1. Finally, the PMMA layer was removed with acetone during the dissolution process, and the device was then annealed at 150 °C for 2 hours.

The fabricated device was inspected and characterized by a field-emission scanning electron microscope (FEI Nova NanoSEM 450) and Raman spectroscopy (Raman Microspectrometer, Takram N-541, Iran). The wavelength of the Raman excitation laser was 532 nm. Current–voltage (*I*–*V*) measurements were performed at room temperature under a DC field using a source unit IVM2.10.15 (Nano Pajouhan Raga Co., Iran).

## 3. Results and discussions

**Fig. 2:**
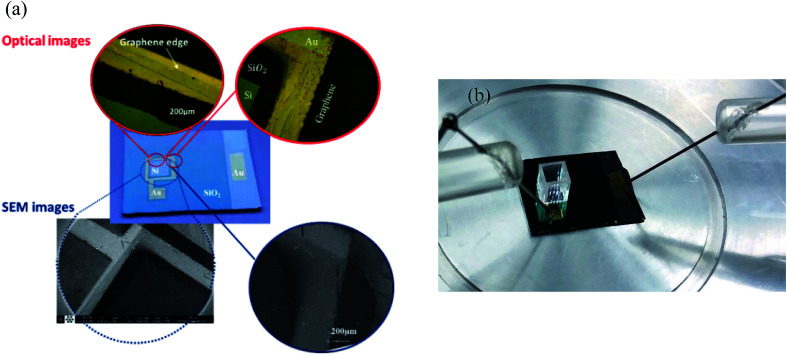
(a) SEM and optical images of the fabricated sensor. Images in the circles show the magnified view of the different parts of the device. (b) An optical image of the measurement setup including the sample with a cuvette placed on the graphene/Si junction.

The scanning electron microscopy (SEM) and optical images of the graphene/Si-based protein sensor are shown in Fig. 2. As shown in this figure, the graphene sheet is connected to the surrounding Au electrode. The Si substrate, SiO_2_ layer, and Au electrodes have been labeled on the optical image. The graphene edge is very clear in the magnified view of the SEM and optical images. The optical image of the measurement setup including the sample with a cuvette placed on the graphene/Si-junction is also shown in part (b) of this figure.

**Fig. 3:**
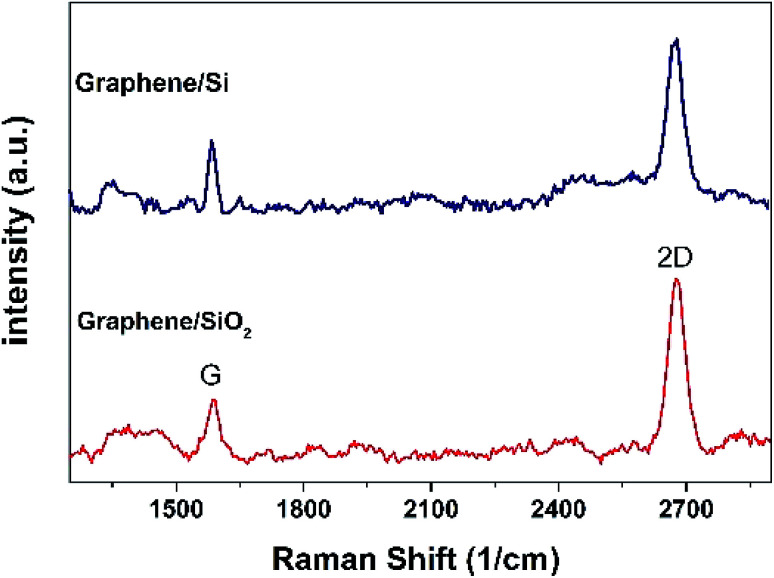
Raman spectrum of the transferred graphene sheet on the Si/SiO_2_ substrate.

The transferred graphene on the Si/SiO_2_ substrate was analyzed by Raman spectroscopy, and the result is shown in Fig. 3. The G-band (1584 cm^−1^) and 2D-band (2673 cm^−1^) Raman peaks of graphene can be seen in this figure. The high peak intensity ratio of 2D to G obtained *via* this analysis indicates high crystallinity and monolayer nature of the graphene sheet.^25^

**Fig. 4:**
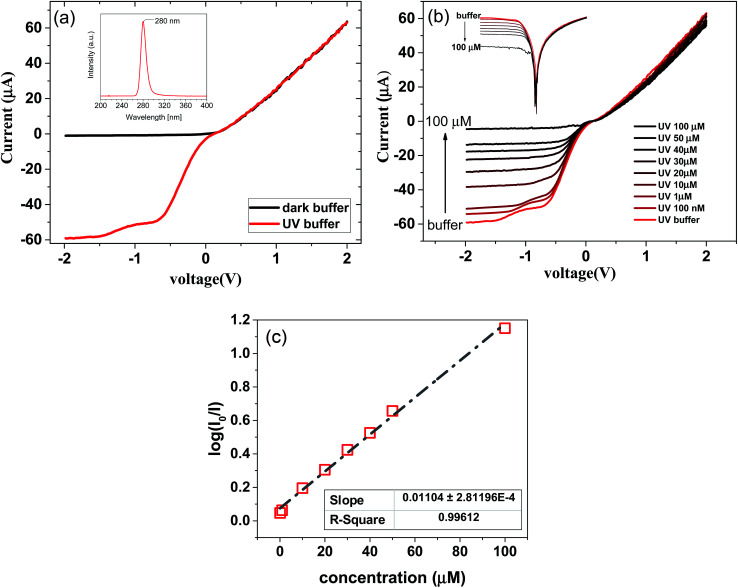
(a) *I*–*V* characteristic of the device in the dark and under UV light irradiation (the surface of the device was covered with the buffer in both measurements). The top inset shows the applied UV light spectra (b). *I*–*V* characteristic of the device under the 280 nm UV light exposure. BSA at different concentrations (100 nM to 100 μM) significantly affects the saturation current of the device. Top inset shows the semi-logarithmic plot. (c) The semi-logarithmic plot of the sensitivity of the device *versus* the BSA concentration. (The voltage bias used for this plot was −1.5 V.)

As abovementioned, the graphene/Si junction behaves as a Schottky diode. Our fabricated device was then utilized for the sensing of BSA *via* monitoring of the electrical behavior of the graphene/Si Schottky junction under UV light irradiation. The electrical behavior of the device in the dark and under UV light irradiation is illustrated in Fig. 4a. The *I*–*V* characteristic of the fabricated device shows a rectifying behavior, confirming the formation of the Schottky junction at the interface of graphene/Si. Fig. 4a demonstrates that exposure of the fabricated device to UV light leads to a significant increment in the reverse bias current of the device. This increment in the reverse bias current due to the light exposure confirms high photo-response of the graphene/Si-based device. The light spectrum of the LED monochromatic UV source used in this experiment is provided in the inset of Fig. 4a.

To apply BSA in a buffer solution onto the device, a small open-base cuvette was directly placed on the device such that the solution covered the entire Si/graphene area. Fig. 4b shows the output characteristic of the device when it was exposed to BSA at different concentrations diluted in phosphate-buffered saline (PBS, pH 7.4) (PBS and BSA were obtained from Sigma-Aldrich, Germany) under UV light exposure. The analyte was exchanged by rinsing the sensor with solvent and blow-drying with nitrogen.

An increase in the BSA concentration leads to a decrement in the forward bias current of the device. This decrement can be explained by considering the electron-donating behavior of BSA that leads to charge concentration difference between the pristine and doped graphene. When graphene was exposed to electron donors, extra electrons were provided, causing an increase in the sheet resistance of graphene. As abovementioned, we have used pH 7.4 for the preparation of different concentrations of BSA. The isoelectric point of BSA is 5.4.^26^ Therefore, BSA behaves as an electron donor on the graphene surface. Moreover, pristine graphene is a p-type semiconductor because of the absorption of moisture or oxygen and PMMA residues. When the graphene surface is exposed to BSA, the Fermi level of graphene is shifted to the Dirac point; this causes an increase in the sheet resistance of graphene. As a result, upon increasing the BSA concentration, the forward current of the junction decreases.

Interestingly, it can be seen that the reverse bias current significantly changes under exposure to BSA at different concentrations. It has been reported that the graphene/Si Schottky junction is highly sensitive in the UVB region.^27^ Thus, UV light exposure leads to an increase in the reverse bias current of the device. When the graphene/Si junction is exposed to UV light, the incident photons generate electron–hole pairs in the structures. By applying a reverse bias, the generated electron–hole pairs are separated, and the injected holes in the graphene lead to a significant photocurrent.^19,28^ On the other hand, when the graphene/Si area is covered with the BSA solution, a decrement in the UV light absorption of the junction occurs because of the absorption of light by albumin. As shown in Fig. 4b, an increase in the BSA concentration leads to a decrease in the reverse bias current of the device. Based on their chemical composition, the chemical species absorb light at a particular wavelength (at 280 nm in the case of BSA). This light absorption obeys the Beer–Lambert law. On the other hand, the optical response of the graphene/Si photodiode is linear.^29^ Accordingly, log(*I*_0_/*I*) *versus* the BSA concentration has a linear response, as shown in the inset of Fig. 4c. The current detection limit in our experiment was 10 nA. According to the results shown in Fig. 4, the detection limit for the BSA protein is about 0.25 nM, which is the same order of magnitude or even better than that of the reported sensors.^30,31^ Note that we did not use any conjugated nanoparticles or markers for sensing; moreover, the sample was not manipulated and could be reused after testing.

The rectifying current–voltage characteristics of the Schottky diode can be expressed by the thermionic emission model in which the diode current can be written as follows:^24^1
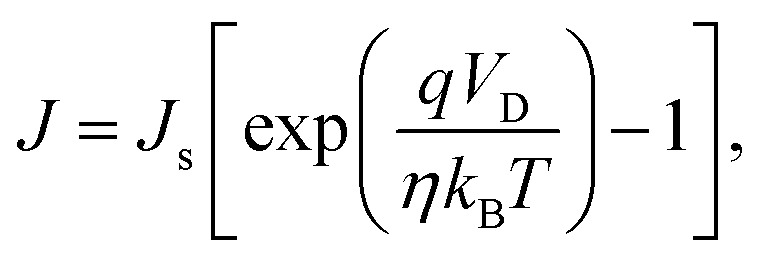
where *η* is the ideality factor, *q* is the electronic charge, *k*_B_ is the Boltzmann constant, *T* is the absolute temperature, and *V*_D_ is the voltage applied across the junction. *V*_D_ can be expressed in the form *V*_D_ = *V* − *IR*_s_, where *R*_s_ is the series resistance. *J*_s_ is defined as the reverse saturation current and can be expressed by2
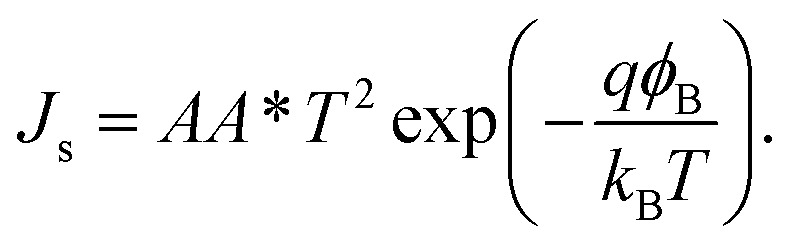
where *A* is the area of the junction, and A* is known as the Richardson constant (112 A cm^−2^ K^−2^ for n-type silicon and 32 A cm^−2^ K^−2^ for p-type silicon). Moreover, *ϕ*_B_ is the Schottky barrier height (SBH). From the result of the *J*–*V* measurement (Fig. 4a) and its analysis with this model, the parameters of the diode were determined. *ϕ*_B_, *η*, series resistance (*R*_s_) and rectification ratio were found to be 0.66 eV, 2.11, 28 kΩ, and 82, respectively.

**Fig. 5:**
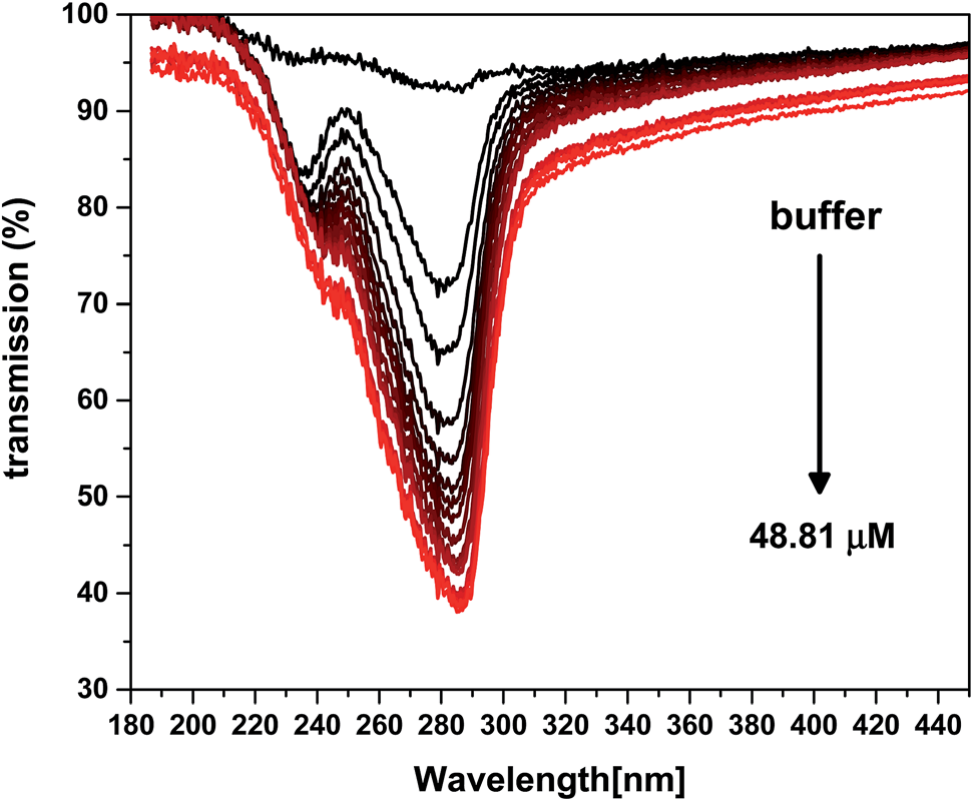
Absorption spectra of BSA at different concentrations.

To monitor the light absorption spectra of BSA, BSA at different concentrations in the buffer solution was examined by a spectrometer (Avantes 2048 spectrometer), and the results are provided in Fig. 5. As shown in this figure, the maximum absorption wavelength of BSA is around 280 nm.

**Fig. 6:**
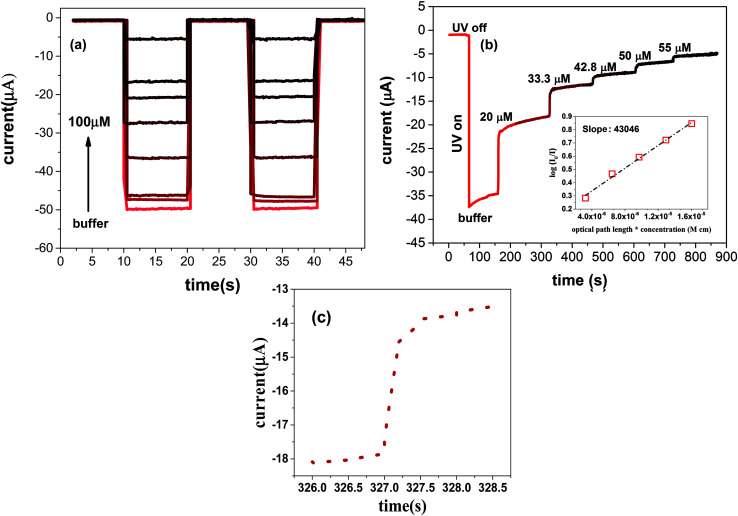
(a) Reverse bias current of the device *versus* time when exposed to BSA at different concentrations. (b) Real-time response of the device to UV light exposure for BSA at different concentrations. (c) Response time for the real time measurement.

The real time measurement of the reverse bias current of the device for different concentration of BSA is plotted in Fig. 6a. Sensitivity as high as 0.5 A M^−1^ obtained by this graphene-based device makes it a promising candidate for accurate measurements in bio-sensing applications. The real-time response of the device to UV light exposure for BSA at different concentrations is also provided in Fig. 6b. The fast response to UV light is a normal behavior of the photodiodes. The rising time for this device is approximately 500 ms (Fig. 6c). An increase in the BSA concentration decreases the photoresponse of the device.

**Fig. 7:**
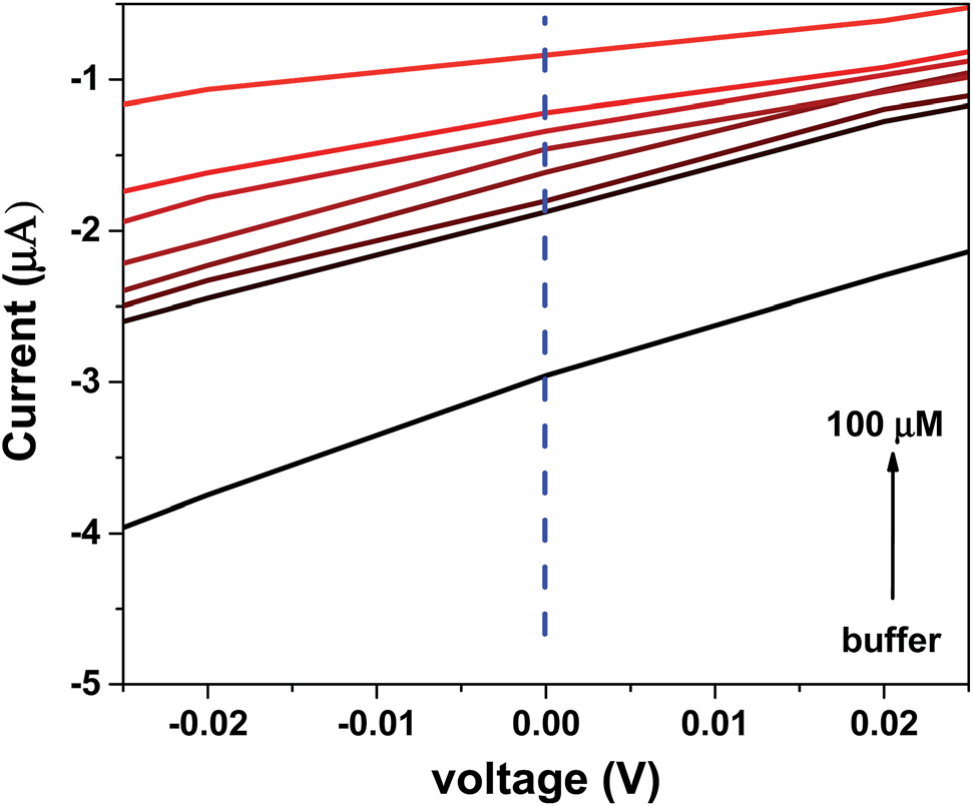
*I*–*V* characteristic of the device under UV light exposure confirming the self-powered response of the device.

Output characteristics of the device near the zero voltage are shown in Fig. 7. As shown in this figure, the fabricated BSA detector has a diode current higher than 3 μA at zero voltage, confirming the self-powered properties of the device that would reduce the need for batteries. It can be seen from this figure that the zero voltage current of the device changes significantly when the device is exposed to BSA at different concentrations.

Moreover, we calculated the responsivity and detectivity of the device for BSA at different concentrations. The responsivity is defined as the ratio of photocurrent to incident optical power irradiated on the device.^32^3
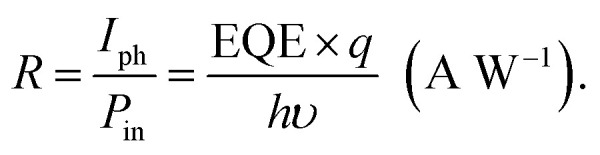


In the eqn (3), EQE, *h*, and *υ* are the external quantum efficiency, Planck's constant and frequency of incident light, respectively. The specific detectivity D* is calculated by the following equation:^33^4
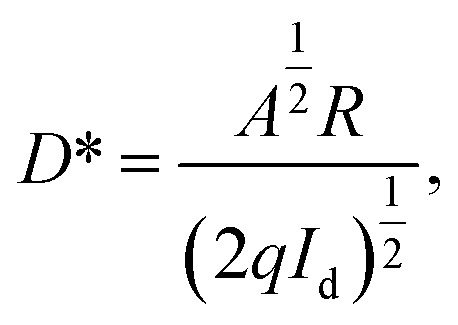
where *I*_d_ represents the dark current density, and *A* is the junction area. For the incident light power of 3.25 mW cm^−2^, dark current of 800 nA and voltage bias of −2 V, the responsivity and detectivity of the device for BSA at different concentrations were calculated, and the results are provided in Table 1. As expected, *R* and D* decrease with the increasing the BSA concentration.

**Table 1:** Responsivity and detectivity for BSA at different concentration at the bias of −2 V and light power of 3.25 mW cm^−2^

	Buffer	100 nM	1 μM	10 μM	20 μM	30 μM	40 μM	50 μM	100 μM
Photocurrent (μA)	59.13	56.87	51.67	39.21	30.01	22.95	18.56	14.76	7.14
Responsivity (mA W^−1^)	202	191	176	134	102	78	64	50	24
Detectivity (cm W^−1^ Hz^1/2^ × 10^10^)	12	11.2	10.3	7.9	6	4.6	3.78	2.95	1.71

Currently, the use of mobile devices for biosensing and the point-of-care testing are the main approaches in the biosensing platform. The use of mobile and small devices, which just need a small sample volume to perform the measurement, for biological analysis offers numerous benefits such as rapid detection, reliable test results and simplicity. The graphene/Si-based device as a low cost, low power, sensitive, mobile and small device is actually the best candidate for protein sensing applications.

## 4. Conclusion

Herein, a graphene/Si-based Schottky diode was successfully fabricated and utilized for sensing BSA in a buffer solution. The device is very sensitive in the UVB region, and BSA effectively absorbs light in the UVB region. Using these features, we proposed a UVB-driven BSA sensor based on the graphene-Si junction. The output characteristic of the device during exposure to BSA showed that an increase in the BSA concentration leads to a decrease in the reverse bias current of the diode significantly. BSA has a significant light absorption around 280 nm. Consequently, when the Si/graphene area is covered by the BSA solution, a decrement in the UV light absorption of the junction occurs, leading to a decrease in the reverse bias current of the device. The sensitivity as high as 0.5 A M^−1^ and the detection limit of 0.25 nM obtained by monitoring the reverse bias current of the fabricated device indicate good performance of the introduced graphene-based diode for bio-sensing applications. In addition, the hybrid graphene-Si exhibits a wide detection range (from 100 nM to 100 μM). The output characteristic of the sensor at the zero bias showed self-powered properties of the device that would reduce the need for batteries. This capability in addition to the simplicity of device fabrication as well as its operating mechanism and high sensitivity is very important in the field of on-chip bio-sensing applications.

## Conflicts of interest

There are no conflicts to declare.

## Supplementary Material
